# Factors affecting HPV infection in U.S. and Beijing females: A modeling study

**DOI:** 10.3389/fpubh.2022.1052210

**Published:** 2022-12-14

**Authors:** Huixia Yang, Yujin Xie, Rui Guan, Yanlan Zhao, Weihua Lv, Ying Liu, Feng Zhu, Huijuan Liu, Xinxiang Guo, Zhen Tang, Haijing Li, Yu Zhong, Bin Zhang, Hong Yu

**Affiliations:** ^1^Labor Model Health Management Center, Beijing Rehabilitation Hospital, Capital Medical University, Beijing, China; ^2^Respiratory Rehabilitation Center, Beijing Rehabilitation Hospital, Capital Medical University, Beijing, China

**Keywords:** human papillomavirus, vaccine, prevalence, NHANES, Beijing, Modelbest, influencing factor

## Abstract

**Background:**

Human papillomavirus (HPV) infection is an important carcinogenic infection highly prevalent among many populations. However, independent influencing factors and predictive models for HPV infection in both U.S. and Beijing females are rarely confirmed. In this study, our first objective was to explore the overlapping HPV infection-related factors in U.S. and Beijing females. Secondly, we aimed to develop an R package for identifying the top-performing prediction models and build the predictive models for HPV infection using this R package.

**Methods:**

This cross-sectional study used data from the 2009–2016 NHANES (a national population-based study) and the 2019 data on Beijing female union workers from various industries. Prevalence, potential influencing factors, and predictive models for HPV infection in both cohorts were explored.

**Results:**

There were 2,259 (NHANES cohort, age: 20–59 years) and 1,593 (Beijing female cohort, age: 20–70 years) participants included in analyses. The HPV infection rate of U.S. NHANES and Beijing females were, respectively 45.73 and 8.22%. The number of male sex partners, marital status, and history of HPV infection were the predominant factors that influenced HPV infection in both NHANES and Beijing female cohorts. However, condom application was not an independent influencing factor for HPV infection in both cohorts. R package Modelbest was established. The nomogram developed based on Modelbest package showed better performance than the nomogram which only included significant factors in multivariate regression analysis.

**Conclusion:**

Collectively, despite the widespread availability of HPV vaccines, HPV infection is still prevalent. Compared with condom promotion, avoidance of multiple sexual partners seems to be more effective for preventing HPV infection. Nomograms developed based on Modelbest can provide improved personalized risk assessment for HPV infection. Our R package Modelbest has potential to be a powerful tool for future predictive model studies.

## Introduction

Human papillomavirus (HPV) infection is an important carcinogenic infection highly prevalent among many populations. It is transmitted *via* sexual intercourse or intimate interpersonal contact (1–3). There are over 200 different HPV types. Different HPV subtypes can cause different pathological lesions, and more than 60 types of HPV are found predominantly or exclusively in the anogenital tract (4).

HPVs can be broadly divided into low- and high-risk types. Low-risk HPVs (e.g., HPV 6 and 11) cause non-malignant lesions (e.g., genital warts and recurrent respiratory papillomatosis). While the high-risk HPVs (e.g., HPV 16 and 18) may lead to various cancers (e.g., nearly all cervical cancers, and most vulvar, vaginal, oropharyngeal, anal, and penile cancers (5)). The oncoproteins E6 and E7 of high-risk HPVs are responsible for HPV oncogenesis (6, 7). The cervical transition zone is extremely vulnerable to HPV infections, and it is the predominant site of origin of cervical cancer (8).

Studies have shown that HPV infection might be associated with sexual behaviors, parity, marital status and age (9–13). However, the relation between condom use and HPV infection remains controversial (13). Exploring HPV-infection associated factors is important for HPV prevention and management. This especially so moving to an era of precision medicine, with artificial intelligence and machine learning finding increasing applications in health care (14–16). Accordingly, prediction models based upon risk factors and/or other parameters, which can serve as a convenient tool for individualized risk estimation as well as risk prevention, have attracted increasing attention (17). Nevertheless, very few studies till date have built a stable and generalizable model for predicting HPV infection risk. Based on the afore-mentioned background, the present study has been executed with two principal goals in mind. One was to identify the overlapping HPV infection-related factors in female populations from the U.S. and Beijing, China. The secondary objective included developing an R package for identifying the top-performing prediction models as well as building predictive models for HPV infection *via* this R package.

## Methods and materials

### Study design and participant selection

This was a retrospective cross-sectional analysis in two cohorts. One cohort is from the National Health and Nutrition Examination Survey (NHANES), which is a national cross-sectional study designed to provide nationally representative data on the nutritional and health status of the non-institutionalized, civilian U.S. population. Data in NHANES were collected through participant interviews and physical examinations. All NHANES study protocols were approved by the National Center for Health Statistics (NCHS) Institutional Review Board and all subjects provided written informed consent before data collection. Detailed descriptions of the methodology and data can be accessed at http://www.cdc.gov/nchs/nhanes/. Since the HPV vaccine was only extensively pursued after 2006 in U.S., we limited our study to female participants in NHANES between 2009 and 2016. The exclusion criteria included: (1) participants aged < 18 or ≥60 years; (2) or with a history of malignancy; (3) or a history of hysterectomy.

Another cohort was from a Beijing Municipal Federation of Trade Unions (BMFTU)-funded cervical screening program for female workers in Beijing. The information needed for this study was sent out to female union members by BMFTU. Females who fulfilled the inclusion/exclusion criteria were invited to take part in this study at Beijing Rehabilitation Hospital. Eligible females were only included as participants in this study after informed consent had been obtained. All participants received questionnaires and HPV genotype testing between April 8, 2020 and May 8, 2020. This study was reviewed and approved by the Ethical Committee of Beijing Rehabilitation Hospital. For this study, the inclusion criteria included: (1) females with a history of having had sex; (2) aged between 20 and 70 years. The exclusion criteria were: (1) history of malignancy; (2) or history of cervical conization or hysterectomy; (3) or history of pelvic radiation therapy; (4) or history of immune disorders; (5) or pregnancy or childbirth within 6 months. For the data analysis of this study, cases with incomplete information were excluded. After completing the questionnaire, the participants received gynecological examinations including HPV genotype test. The detailed procedure of the HPV examination has been described in our previous study (18). Briefly, cervical cells of each participant were sampled using a cervix brush, placed in a vial containing 3 mL of isotonic sodium chloride and then stored at 4°C. Fifteen HPV genotypes included 13 high-risk genotypes (HPV 16, 18, 31, 33, 35, 39, 45, 51, 52, 56, 58, 59, and 68) and two low-risk genotypes (HPV 6 and 11) were examined. This HPV testing method can detect concentrations as low as 1.0 × 10^3^ copies/mL of HPV, and the specificity is more than 98%. Detailed content of the questionnaire and the HPV test are shown in Supplementary Table 1.

### Cohort definition and feature evaluation

For the NHANES cohort, the participants were randomly divided in a 7:3 ratio into the training and test-evaluation sets (the test-evaluation set was further divided to test and evaluation sets at a ratio of 2:1) using the “sample” function in R software (version 4.1.3). The training and test sets were used to select features and develop the prediction model. The evaluation set was used to evaluate the performance of prediction model. We also evaluated the prediction model in the combined test-evaluation set. Dividing the data into three data sets (rather than the training-validation binary divisions) could avoid model overfitting and provide an approach to validate the model (19).

For the feature evaluation, LASSO and multivariate logistic regression analysis were performed successively *via* R packages glmnet (v.4.1-3) and rms (v.6.2-0) on the training set. Features with *P*-values < 0.05 in both LASSO and multivariate logistic regression analysis were regarded as independent factors associated with HPV infection. R package forestplot (v.2.0.1) was used to graphically represent results of multivariate logistic regression analysis. Same methods were also performed on the Beijing female cohort.

### Method and principle of the R package modelbest

**Figure 1:**
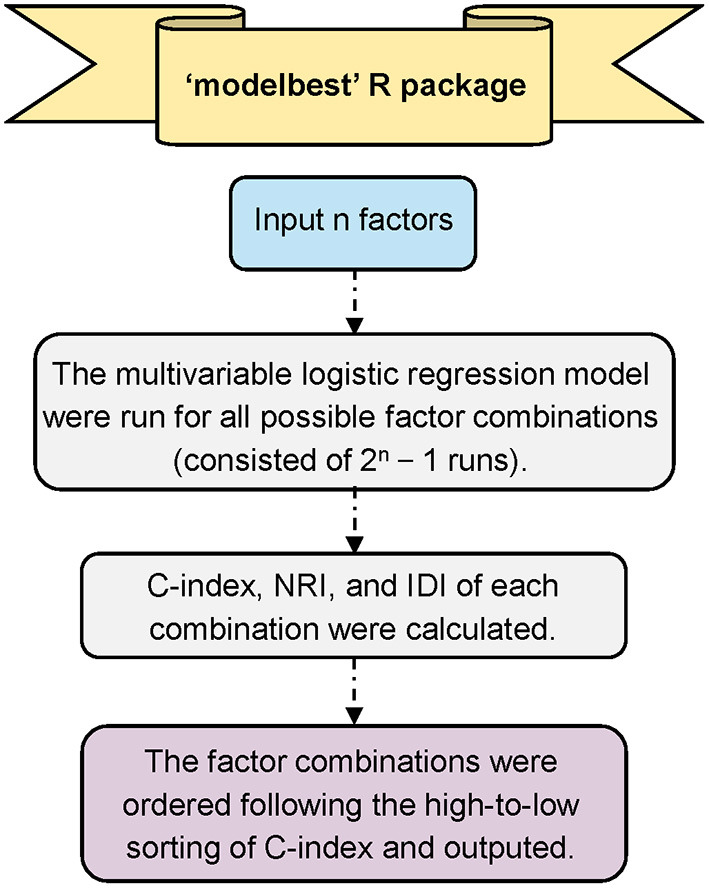
Overall workflow of Modelbest. C-index, Harrells concordance index; NRI, net reclassification index; IDI, integrated discrimination index.

The Modelbest package (https://github.com/yhx68/best/blob/main/Modelbest_0.1.0.tar.gz) iterates through all possible variables' combinations in a multivariate logistic regression model and outputs all models ordered following high-to-low sorted order based on the Harrells concordance index (C-index); meanwhile, this package can automatically identify the statistically significant variables in multivariate logistic regression among the input variables, and calculate the net reclassification index (NRI) and integrated discrimination index (IDI) of each model compared with the baseline prediction model (i.e., the model that contains only the significant variables in multivariate logistic regression analysis) (Figure 1). The output results include the model formula, number of factors in the model, C-index, NRI and IDI, etc. For the input file of Modelbest, all variables need to be categorical variables. The first column is always the outcome variable, and the following columns are always the candidate factors for model construction. The candidate factors can be the statistically significant variables in logistic regression analysis, beyond that, factors deemed clinically important can also be considered.

The C-index has a range from 0.5 to 1.0, 0.5 represents random prediction, while 1.0 indicates perfect prediction. IDI and NRI are newer techniques to quantify the improvement of a new model's predictive performance over a baseline prediction model (20). NRI and IDI values larger than zero indicate the new model has better performance than the baseline prediction model.

### Nomogram construction and evaluation

The consensus guidelines (i.e., the number of covariates per model was limited by the number of events, and at least 10 events were required for each covariate) (21–23) were followed in constructing the prediction models for HPV infection. To avoid potentially important variables being missed, the Modelbest package was applied to identify the top-performing predictive model for HPV infection. Variables included in the Modelbest package were statistically significant variables in the LASSO regression analysis. Results of Modelbest package are shown in Supplementary Table 2 (for NHANES cohort) and Supplementary Table 3 (for Beijing female cohort). The variables' combination with the highest C-index, NRI, and IDI was selected for constructing nomogram. Rule of thumb for multivariable models (i.e., at least 10 outcome events per variable) was considered when choosing the best combination. R packages rms (v.6.2-0) and regplot (v. 1.1) were applied to obtain nomogram.

To evaluate the nomogram's discrimination ability, the receiver operating characteristic (ROC) plot and area under ROC curve (AUC), C-index, NRI, IDI, calibration curves and decision curve analysis (DCA) were performed in R using rms (v.6.2-0), ROCR (v. 1.0-11), Hmisc (v. 4.6-0), PredictABEL (v. 1.2-4) and rmda (v. 1.6) packages. Finally, online calculators for HPV infection were constructed *via* R packages DynNom (v. 5.0.1), shiny (v. 1.7.1) and rsconnect (v. 0.8.25). All analysis in this study was conducted using R software (version 4.1.3). Supplementary Table 4 summarizes the R packages used in this study and the corresponding analysis.

## Results

### Participant characteristics

**Figure 2:**
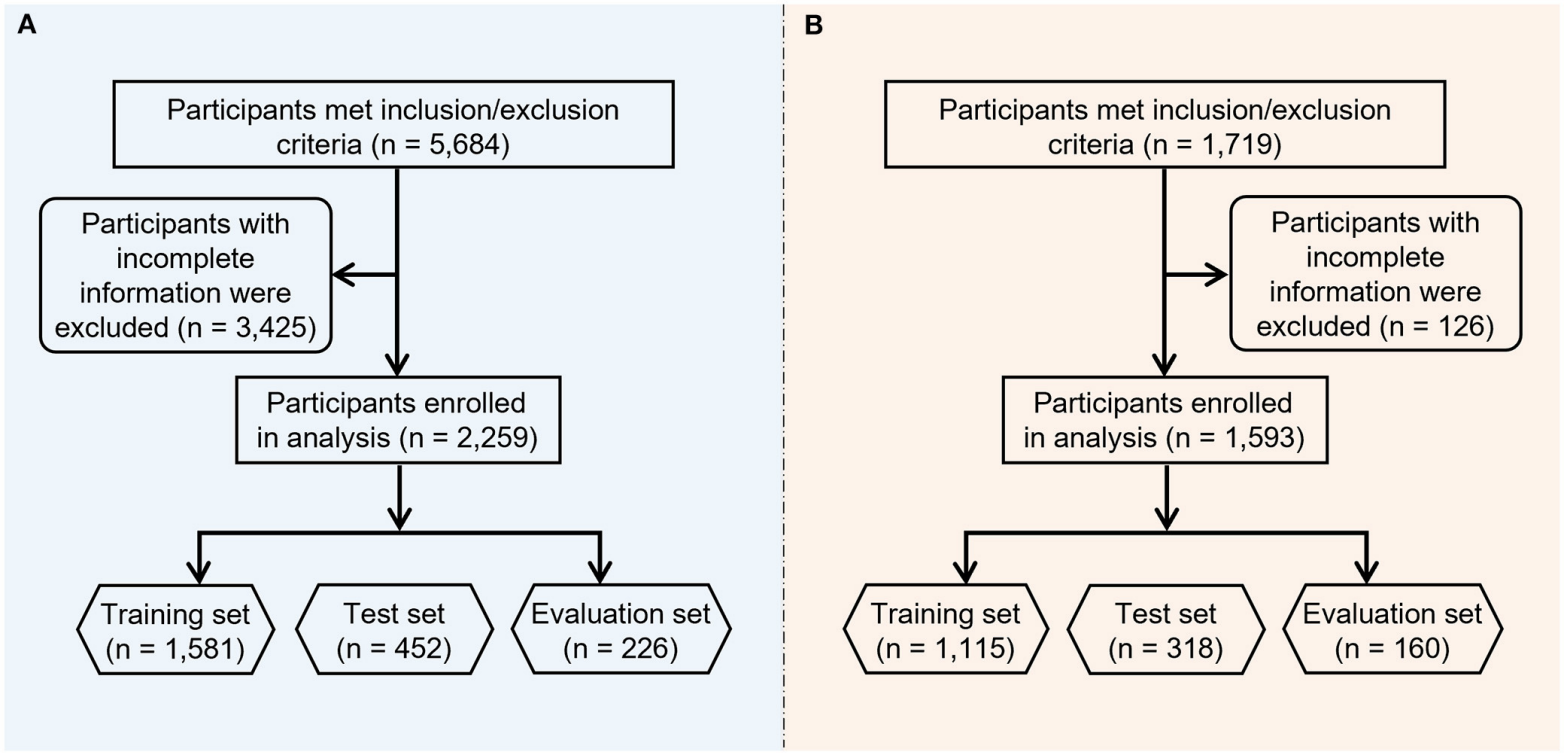
Study flow of participants from **(A)** NHANES cohort and **(B)** Beijing female cohort.

The study flowchart is shown in Figure 2. Primary and secondary objectives were fulfilled. For the NHANES cohort of 2009–2016, a total of 5,684 participants met the inclusion/exclusion criteria (see methods section), following the exclusion of 3,425 participants with incomplete information, 2,259 participants were included in the final analysis. Among these 2,259 participants, 45.73% (1,033/2,259) were HPV positive.

For the Beijing female cohort, 1,719 female union workers from various industries participated in this program, following the exclusion of 126 participants with missing data, 1,593 female workers in total were included in the current study's analysis. These participants came from the security industry, governmental agencies, cleaning departments, construction industry, transportation industry, education industry, express delivery industry, property sector and medical industry. Among these 1,593 participants, 8.22% (131/1,593) were HPV positive. Supplementary Tables 5, 6 summarize the general characteristics of the NHANES and Beijing female cohorts.

### Factors associated with HPV infection

**Figure 3:**
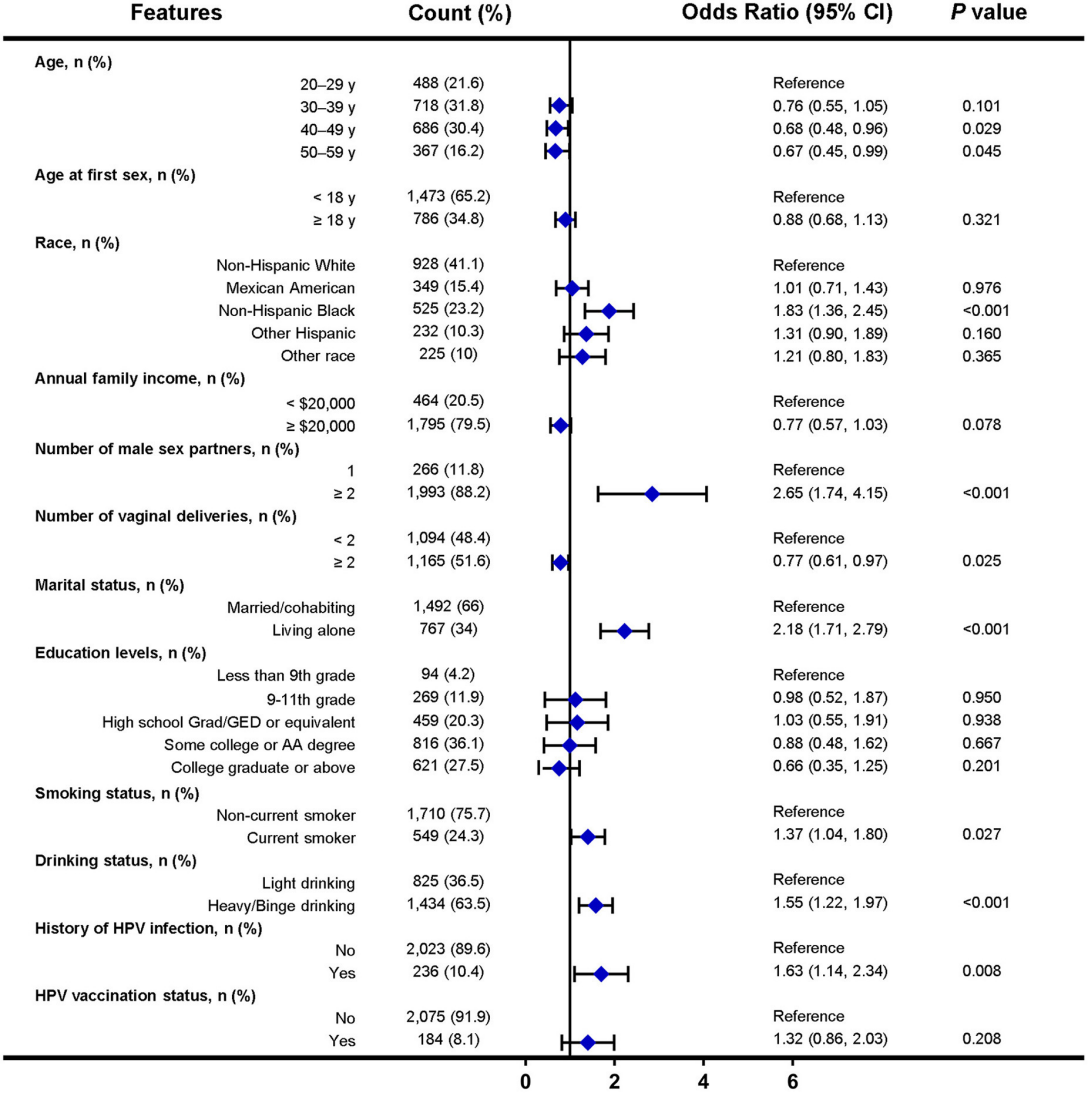
Forest plot of multivariable logistic regression analysis in the training set of NHANES cohort. The x-axis represents the odds ratio scale. An odds ratio >1 indicates increased odds of HPV infection, whereas an odds ratio < 1 suggests decreased odds of HPV infection. CI, confidence interval.

In the NHANES cohort, 12 factors were selected for multivariate logistic analysis after LASSO regression, and eight factors (including age, race, number of male sex partners, number of vaginal deliveries, marital status, smoking status, drinking status, and history of HPV infection) were found to be independent factors significantly associated with HPV infection (Figure 3). Notably, factors such as condom use, menstruation status, educational levels, sleep disorder history, chlamydia infection history, and daily sedentary activity were not significantly associated with HPV infection.

**Figure 4:**
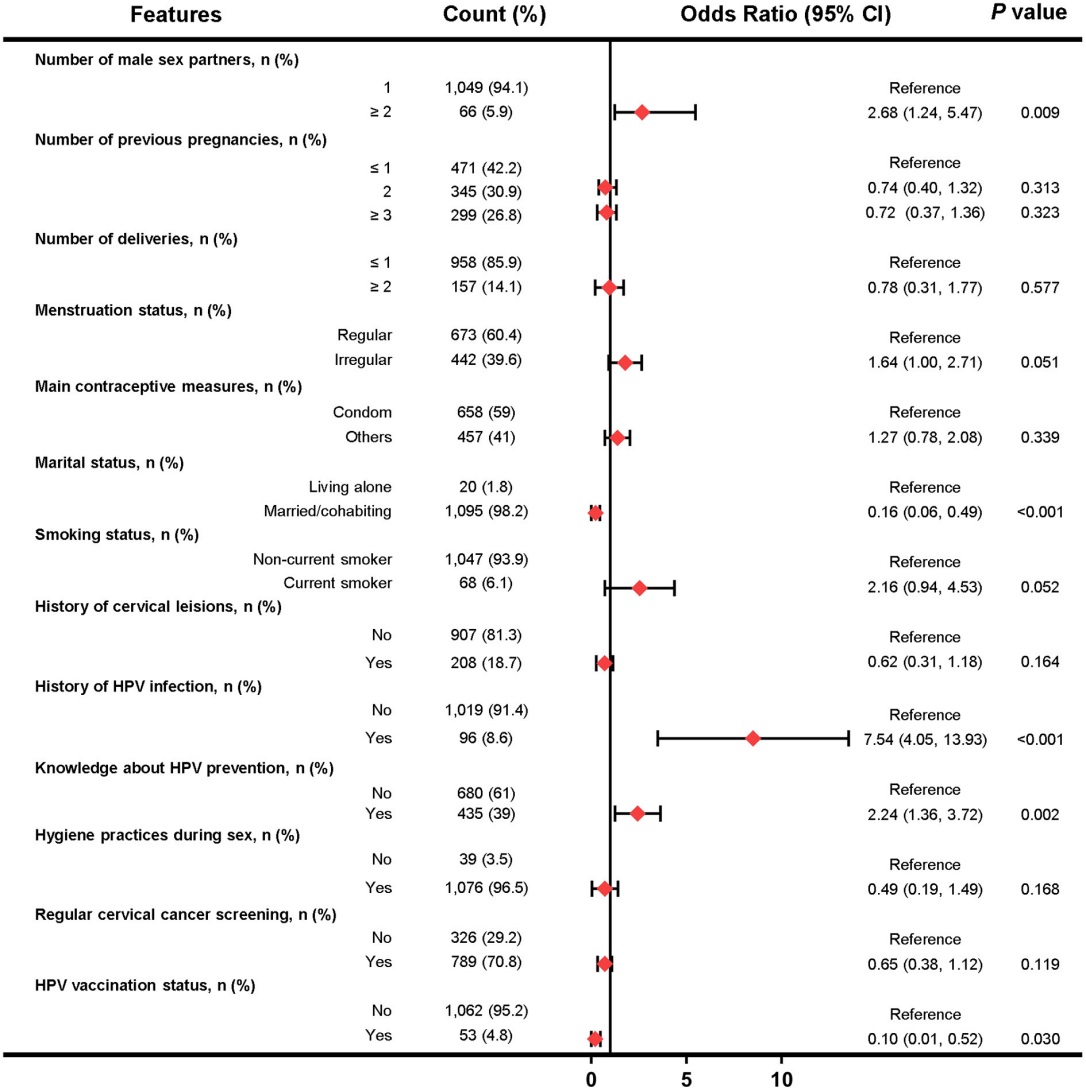
Forest plot of multivariable logistic regression analysis in the training set of Beijing female cohort. The x-axis represents the odds ratio scale. An odds ratio >1 indicates increased odds of HPV infection, whereas an odds ratio < 1 suggests decreased odds of HPV infection.

In the Beijing female cohort, 13 factors were selected for multivariate logistic analysis after LASSO regression, and five factors (including number of male sex partners, marital status, history of HPV infection, knowledge about HPV prevention and HPV vaccination status) were found to be independently associated with HPV infection (Figure 4). However, factors such as condom use, menstruation status and educational levels were not significantly associated with HPV infection.

### Modelbest-models show better performance than log-models for predicting HPV infection

Based on the independently associated factors, the predictive models for HPV infection were constructed (namely log-model). Besides, predictive models based on R package Modelbest were also constructed (namely Modelbest-model). The C-index of the log-models were, respectively 0.792 and 0.691 for the NHANES and Beijing female evaluation sets. While the C-index of the Modelbest-models were, respectively 0.812 and 0.747 for the NHANES and Beijing female evaluation sets. Table 1 shows the NRI and IDI of Modelbest-models than the log-models. The ROC, calibration, and DCA curves of the prediction models (log-models and Modelbest-models) in the NHANES and Beijing female cohorts are shown in Supplementary Figures 1–4.

**Table 1:** NRI and IDI of the Modelbest-developed models for predicting HPV infection than models developed on multivariate logistic regression.

	**Continuous NRI**		**IDI**	
	**OR (95%CI)**	**P-value**	**OR (95%CI)**	**P-value**
NHANES training set	0.128 (0.029–0.227)	0.011	0.008 (0.003–0.012)	< 0.001
NHANES test-evaluation set	0.208 (0.057–0.359)	0.007	0.008 (0.001–0.015)	0.018
NHANES test set	0.234 (0.049–0.418)	0.013	0.013 (0.003–0.024)	0.013
NHANES evaluation set	0.328 (0.076– 0.581)	0.011	0.038 (0.013–0.062)	0.003
Beijing female training set	0.325 (0.104–0.545)	0.004	0.013 (−0.001–0.026)	0.062
Beijing female test-evaluation set	0.325 (0.040–0.610)	0.025	0.019 (−0.002–0.040)	0.078
Beijing female test set	0.271 (-0.007–0.549)	0.056	0.013 (−0.010–0.036)	0.261
Beijing female evaluation set	0.414 (-0.143–0.972)	0.145	0.036 (−0.029–0.101)	0.281

OR, odds ratio; NRI, net reclassification index; IDI, integrated discrimination index; CI, confident interval.

**Figure 5:**
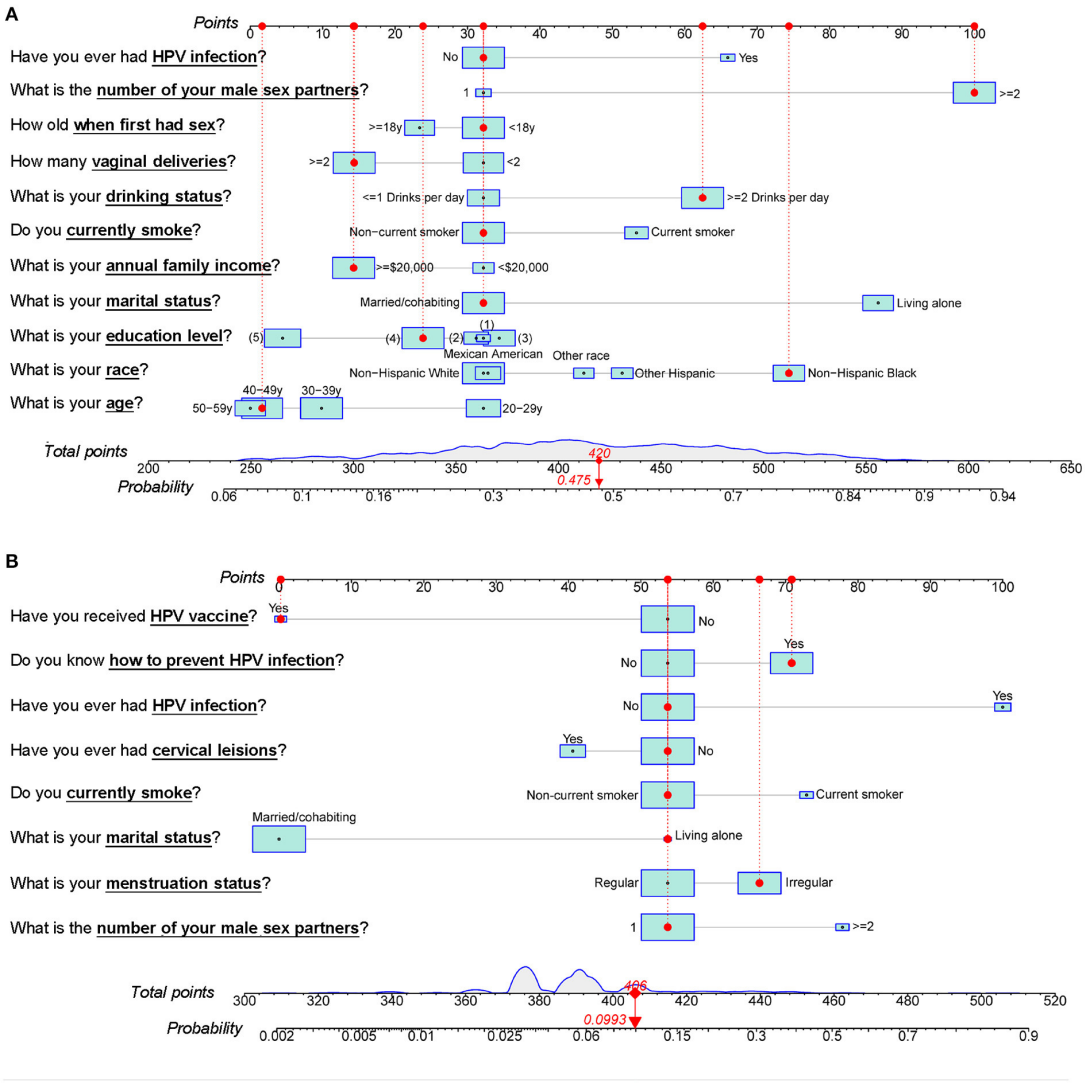
Modelbest-based nomograms for the **(A)** NHANES cohort and **(B)** Beijing female cohort. The red dots, dotted lines, upper and lower red numbers, respectively represent the characteristics, the corresponding points, total points and the likelihood to be infected by HPV for an individual. (1), < 9th Grade. (2), 9–11th Grade (Including 12th grade with no diploma). (3), High School Grad/GED or Equivalent. (4), Some College or an AA degree. (5), College Graduate or above level of education.

Figure 5 contains nomograms constructed based on the Modelbest-models for NHANES and Beijing female cohorts. We also constructed online calculators based on the Modelbest-models for predicting probability of HPV infection (https://cervixuteri.shinyapps.io/HPV1/ is for NHANES cohort; https://cervixuteri.shinyapps.io/HPV2/ is for Beijing female cohort).

## Discussion

Taken together, we found number of male sex partners, marital status, and history of HPV infection were factors significantly associated with HPV infection in both the NHANES and Beijing females. Besides, the NHANES cohort revealed that smoking and drinking status were also independently associated with HPV infection. Interestingly, HPV vaccination status in the NHANES cohort showed no significant association with HPV infection. Based on the R package Modelbest developed in this study and the data of NHANES and Beijing females, we built two prediction models, respectively targeted for NHANES and Beijing females, these two models exhibited good prediction performance for HPV infection.

It is well known that HPV is transmitted predominately through sexual contact, and our findings (i.e., multiple sex partners was independent risk factor for HPV infection in multivariate logistic regression analysis) strongly supported this theory. Earlier studies indicated that two-thirds of women were infected with HPV within 2 years of onset of sexual activity and young women with multiple sex partners are at highest risk of HPV infection (24, 25). Considering the first sexual relationship carries a substantial risk (26, 27), sex education is therefore particularly important for preventing HPV infections. To reduce risk of HPV infection, there is a clear need to provide the general public and especially teenagers with sex education and strategies (such as delayed sexual debut, minimizing the number of sexual partners, and steering away from promiscuous male partners) to prevent HPV infection. Moreover, compared with married/cohabiting women, women living alone may have a higher chance to have a risky sexual partner or multiple sexual partners, therefore, the marital status is also a factor significantly associated with HPV infection in this study. Despite consistent condom use being an effective means against HPV infection, HPV can be transmitted in the genital region through non-penetrative sexual contact (28). Both the NHANES and Beijing female cohorts revealed no significant association between condom use with the probability of cervical HPV infection in the current study. We guess that for the people with high-risk sexual behaviors, the protective effect of condoms against HPV was limited. However, considering the data of the current two cohorts were all retrospective, with the potential risk of confounding and selection bias. Future prospective studies with larger sample sizes are needed to validate this finding (i.e., non-significant association between condom use and HPV infection).

The history of HPV infection was also an independent risk factor for HPV infection in both the NHANES and Beijing female cohorts. It is undeniable that, for most people, HPV infection is transient and can be cleared or become undetectable after 1–2 years of infection (29, 30). Nevertheless, in some instances, HPV infection can be latent and remain at risk for re-activation. This means that women who have ever been infected with HPV (either with or without HPV-vaccination) should pay sufficient attention to HPV and participate in regular re-examinations for HPV.

For the NHANES female cohort, we also found that smoking status and drinking status were the independent risk factors for HPV infection. These findings are in accordance with the previous studies. Smoking has been confirmed in relation to HPV infection (including the HPV incidence, prevalence, persistence, and DNA load) in mounting studies (31–33). Recent study revealed tobacco smoke may interact synergistically with HPV by promoting HPV replication, host DNA damage, HPV E6/E7 expression, and disrupting host immune response against HPV (34). Alcohol drinking behaviors have also been suggested associated with risk of HPV persistence (35). Moreover, another study in U.S. revealed, binge drinkers from young adults were more likely to have HIV/HPV co-infection high-risk behaviors (36).

In the Beijing female cohort, the smoking status showed no significant association with HPV infection in the multivariate logistic regression analysis, this may be partially due to the small sample size, since only 6.10% of the participants (68/1,115) in the training set were current-smokers. In comparison, in the NHANES training set, 24.10% participants (430/1,785) were current-smokers. The statistical results of the Beijing female cohort would probably be more convincing if the sample size was increased. In the Beijing female cohort, we also found that knowledge about HPV prevention was a risk factor for HPV infection. To some extent, this may reflect the lack of public education about HPV in China, as plenty of women learned about HPV and the HPV prevention strategies after the diagnosis of HPV infections.

Based on the NHANES females, we observed no significant correlation between HPV infection and vaccination. This may be due to the HPV vaccine being a newer vaccine with limited application time. The Food and Drug Administration (FDA) only licensed the first HPV vaccine on June, 2006 for females 9–26 years, and the most recent nine-valent HPV vaccine was not approved by the FDA until 2014. Some women may have been infected with HPV prior to HPV vaccination, nevertheless, the vaccine is not therapeutic and may only prevent future infection. In addition, the Roche HPV linear array applied in the NHANES females can detect 37 HPV types, but currently available HPV vaccines can prevent at most of nine types of high-risk HPV infection. It is also possible that a few women did not develop effective immunity response after vaccination. This suggests that, for sexually active women, HPV vaccination is only part of a successful strategy for preventing HPV infection and cervical cancer, and that regular cervical screening and safer sex approaches still play an integral role.

Nomograms, as a reliable and convenient tool for individual risk prediction, have been widely used in the medical field and for guiding clinical decision (37, 38). The C-index and AUC are the main reference for evaluating the predictive performance of a nomogram. Typically, AUC and C-index values larger than 0.7 are considered acceptable discrimination. Additionally, other methods such as calibration and DCA curves, NRI and IDI have also been applied for evaluating the reliability and improvements of the nomogram. To date, there is no uniform screening standard for variables incorporated into the clinical prediction model, but it is recommended to consider both statistically significant results and universally accepted clinical knowledge when constructing a clinical prediction model (39–41). The R package Modelbest developed in this study could help researchers evaluate the performance of every possible combination of variables and choose the best performing combination based on objective evaluation indicators (C-index, NRI, and IDI). Based on the data of NHANES and Beijing female cohorts, we developed nomograms for predicting HPV infection using R package Modelbest. Modelbest-based predictive models showed better performance than the models developed only based on significant variables in multivariate logistic regression analysis. To our knowledge, this is the first study to predict HPV infection through nomograms and, in addition, to establish a powerful R package for future predictive model studies.

However, there are several limitations to this study: First, its main limitation is that it is retrospective. Second, the predictive models built in this study lack external validation. For example, the research on the Beijing female cohort was conducted among Beijing female union workers who live in areas with a great availability of medical services and are provided ThinPrep cytology test and HPV testing yearly. This characteristic of the cohort limits the generalizability of the Beijing-female-based predictive model to other regions/populations. In comparison, the NHANES are a series of nationally representative cross-sectional surveys for community-dwelling U.S. population using a complex multistage probability sampling design, which allows for generalizability of the NHANES-based predictive model to the U.S. population. Third, the study covers a small sample size, which may lead to some factors being insignificant in the present study. The heterogeneity in study design and populations between NHANES and Beijing female cohorts is another limitation, which was partly reflected in the differences in HPV detection methodologies (i.e., HPV genotype testing in NHANES cohort could detect 37 types of HPV, while testing in the Beijing female cohort could detect 15 types). The enormous population heterogeneity prevents any standardization of data across these two populations. However, this study was not designed to compare the HPV infection rates between different cohorts, but to find the overlapping risk factors for HPV infection among different regions and construct targeted prediction models for different regions.

## Conclusion

Collectively, in this study, we found number of male sex partners, marital status and history of HPV infection were significantly correlated with HPV infection in both NHANES and Beijing female cohorts, and the multi-cohort findings increase the credibility and generalizability of our conclusion. Beyond that, we developed an R package (Modelbest) which could serve as a powerful tool for identifying the top-performing prediction models in future studies. The Modelbest-developed nomograms in this study showed good predictive performance and can provide risk assessments for HPV infection that are both personalized and evidence-based.

## Data availability statement

The raw data supporting the conclusions of this article will be made available by the authors, without undue reservation.

## Ethics statement

The study involving Beijing female participants was reviewed and approved by the Ethical Committee of Beijing Rehabilitation Hospital. The participants provided their written informed consent to participate in this study. Ethics approval and informed consent were not required for the analysis of the publicly available NHANES data.

## Author contributions

HYu, BZ, YZho, and HYa designed the research. YZha performed HPV testing. WL, YL, FZ, XG, ZT, and HLi conducted questionnaire survey. HYa, YX, RG, and HLiu analyzed data and developed R package Modelbest. HYa wrote the manuscript. HYu, BZ, and YZho proofread and revised the manuscript. All authors contributed to the article and approved submitted version.

## References

[1] Schottenfeld D Beebe-Dimmer JL. Advances in cancer epidemiology: Understanding causal mechanisms and the evidence for implementing interventions. Annu Rev Public Health. (2005) 26:37–60. 10.1146/annurev.publhealth.26.021304.144402 15760280

[2] Zimet GD Shew ML Kahn JA. Appropriate use of cervical cancer vaccine. Annu Rev Med. (2008) 59:223–36. 10.1146/annurev.med.59.092806.131644 18186704

[3] Ardekani A Taherifard E Mollalo A Hemadi E Roshanshad A Fereidooni R. Human papillomavirus infection during pregnancy and childhood: a comprehensive review. Microorganisms. (2022) 10:1932. 10.3390/microorganisms10101932 36296208 PMC9607260

[4] Alarcón-Romero LDC Organista-Nava J Gómez-Gómez Y Ortiz-Ortiz J Hernández-Sotelo D Del Moral-Hernández O. Prevalence and distribution of human papillomavirus genotypes (1997–2019) and their association with cervical cancer and precursor lesions in women from Southern Mexico. Cancer Control. (2022) 29:10732748221103331. 10.1177/10732748221103331 35608056 PMC9136461

[5] Massarelli E William W Johnson F Kies M Ferrarotto R Guo M. Combining immune checkpoint blockade and tumor-specific vaccine for patients with incurable human papillomavirus 16-related cancer: a phase 2 clinical trial. JAMA Oncol. (2019) 5:67–73. 10.1001/jamaoncol.2018.4051 30267032 PMC6439768

[6] Kiran S Dar A Singh SK Lee KY Dutta A. The deubiquitinase USP46 is essential for proliferation and tumor growth of HPV-transformed cancers. Mol Cell. (2018) 72:823–835.e5. 10.1016/j.molcel.2018.09.019 30415951 PMC6294304

[7] García A Maldonado G González JL Svitkin Y Cantú D García-Carrancá A. High-risk human papillomavirus-18 uses an mRNA sequence to synthesize oncoprotein E6 in tumors. Proc Natl Acad Sci U S A. (2021) 118:e2108359118. 10.1073/pnas.2108359118 34615711 PMC8522272

[8] Chumduri C Gurumurthy RK Berger H Dietrich O Kumar N Koster S. Opposing Wnt signals regulate cervical squamocolumnar homeostasis and emergence of metaplasia. Nat Cell Biol. (2021) 23:184–97. 10.1038/s41556-020-00619-0 33462395 PMC7878191

[9] Vaccarella S Herrero R Dai M Snijders PJF Meijer CJLM Thomas JO. Reproductive factors, oral contraceptive use, and human papillomavirus infection: Pooled analysis of the IARC HPV prevalence surveys. Cancer Epidemiol Biomarkers Prev. (2006) 15:2148–53. 10.1158/1055-9965.EPI-06-0556 17119039

[10] Sierra MS Tsang SH Hu S Porras C Herrero R Kreimer AR. Risk factors for non-human papillomavirus (HPV) type 16/18 cervical infections and associated lesions among HPV DNA-negative women vaccinated against HPV-16/18 in the Costa Rica vaccine trial. J Infect Dis. (2021) 224:503–16. 10.1093/infdis/jiaa768 33326576 PMC8496490

[11] Mchome BL Kjaer SK Manongi R Swai P Waldstroem M Iftner T. types, cervical high-grade lesions and risk factors for oncogenic human papillomavirus infection among 3416 Tanzanian women. Sex Transm Infect. (2021) 97:56–62. 10.1136/sextrans-2019-054263 32269071

[12] Castellsagué X Bosch FX Muñoz N. Environmental co-factors in HPV carcinogenesis. Virus Res. (2002). p. 191–199 10.1016/S0168-1702(02)00188-0 12445659

[13] Chelimo C Wouldes TA Cameron LD Elwood JM. Risk factors for and prevention of human papillomaviruses (HPV), genital warts and cervical cancer. J Infect. (2013) 66:207–17. 10.1016/j.jinf.2012.10.024 23103285

[14] Beam AL Kohane IS. Big data and machine learning in health care. JAMA J Am Med Assoc. (2018) 319:1317–8. 10.1001/jama.2017.18391 29532063

[15] Han R Cheng G Zhang B Yang J Yuan M Yang D. Validating automated eye disease screening AI algorithm in community and in-hospital scenarios. Front Public Heal. (2022) 10:944967. 10.3389/fpubh.2022.944967 35937211 PMC9354491

[16] Li F Pan J Yang D Wu J Ou Y Li H. A multicenter clinical study of the automated fundus screening algorithm. Transl Vis Sci Technol. (2022) 11:22–22. 10.1167/tvst.11.7.22 35881410 PMC9339691

[17] Holmer R,. Developing an HPV Infection Risk Prediction Model for Adult Females. Industrial Engineering Undergraduate Honors Theses. (2018). Available online at: https://scholarworks.uark.edu/ineguht/55 (accessed November 20, 2022).

[18] Yang HX Zhong Y Lv WH Yu H. Factors associated with human papillomavirus infection - Findings from a cervical cancer screening program for female employees in Beijing. Cancer Manag Res. (2019) 11:8033–41. 10.2147/CMAR.S209322 31695489 PMC6717858

[19] Goldstein BA Navar AM Carter RE. Moving beyond regression techniques in cardiovascular risk prediction: applying machine learning to address analytic challenges. Eur Heart J. (2017) 38:1805–14. 10.1093/eurheartj/ehw302 27436868 PMC5837244

[20] Cook NR. Statistical evaluation of prognostic vs. diagnostic models: beyond the ROC curve. Clin Chem. (2008) 54:17–23. 10.1373/clinchem.2007.096529 18024533

[21] Kleinbaum D Klein M. Logistic Regression: A self-Learning text (Statistics for Biology and Health). New York: Springer-Verlag. (2002).

[22] Vittinghoff E McCulloch CE. Relaxing the rule of ten events per variable in logistic and cox regression. Am J Epidemiol. (2007) 165:710–8. 10.1093/aje/kwk052 17182981

[23] Harrel Jr. FE. Regression modeling strategies—with applications to linear models, logistic and ordinal regression, and survival analysis. R Softw. (2015) 70:598. 10.1007/978-3-319-19425-7

[24] Koutsky L. Epidemiology of genital human papillomavirus infection. Am J Med. (1997) 102:3–8. 10.1016/S0002-9343(97)00177-0 9217656

[25] Couture MC Page K Stein ES Sansothy N Sichan K Kaldor J. Cervical human papillomavirus infection among young women engaged in sex work in Phnom Penh, Cambodia: prevalence, genotypes, risk factors and association with HIV infection. BMC Infect Dis. (2012) 12:1–11. 10.1186/1471-2334-12-166 22839728 PMC3436768

[26] Kim DK Hunter P. Recommended adult immunization. Ann Intern Med. (2020) 172:337–47. 10.7326/M20-0046 32016359 PMC9555290

[27] Winer RL Feng Q Hughes JP O'Reilly S Kiviat NB Koutsky LA. Risk of female human papillomavirus acquisition associated with first male sex partner. J Infect Dis. (2008) 197:279–82. 10.1086/524875 18179386 PMC2875685

[28] Liu Z Rashid T Nyitray AG. Penises not required: a systematic review of the potential for human papillomavirus horizontal transmission that is non-sexual or does not include penile penetration. Sex Health. (2016) 13:10–21. 10.1071/SH15089 26433493

[29] Schiffman M Castle PE Jeronimo J Rodriguez AC Wacholder S. Human papillomavirus and cervical cancer. Lancet. (2007) 370:890–907. 10.1016/S0140-6736(07)61416-0 17826171

[30] Ranjeva SL Baskerville EB Dukic V Villa LL Lazcano-Ponce E Giuliano AR. Recurring infection with ecologically distinct HPV types can explain high prevalence and diversity. Proc Natl Acad Sci USA. (2017) 114:13573–8. 10.1073/pnas.1714712114 29208707 PMC5754802

[31] Umutoni V Schabath MB Nyitray AG Wilkin TJ Villa LL Lazcano-Ponce E. The association between smoking and anal human papillomavirus in the HPV infection in men study. Cancer Epidemiol Biomarkers Prev. (2022) 31:1546–53. 10.1158/1055-9965.EPI-21-1373 35653709 PMC9350906

[32] Xi LF Koutsky LA Castle PE Edelstein ZR Meyers C Ho J. Cigarette smoking linked to increased human papillomavirus DNA load. CA Cancer J Clin. (2010) 60:137–38. 10.3322/caac.20072 20375321

[33] Xi LF Koutsky LA Castle PE Edelstein ZR Meyers C Ho J. Relationship between cigarette smoking and human papilloma virus types 16 and 18 DNA load. Cancer Epidemiol Biomarkers Prev. (2009) 18:3490–6. 10.1158/1055-9965.EPI-09-0763 19959700 PMC2920639

[34] Aguayo F Muñoz JP Perez-Dominguez F Carrillo-Beltrán D Oliva C Calaf GM. High-risk human papillomavirus and tobacco smoke interactions in epithelial carcinogenesis. Cancers. (2020) 12:2201. 10.3390/cancers12082201 32781676 PMC7465661

[35] Oh HY Kim MK Seo S Lee DO Chung YK Lim MC. Alcohol consumption and persistent infection of high-risk human papillomavirus. Epidemiol Infect. (2015) 143:1442–50. 10.1017/S0950268814002258 25185457 PMC9507201

[36] Olusanya OO Wigfall LT Rossheim ME Tomar A Barry AE. Binge drinking, HIV/HPV co-infection risk, and HIV testing: factors associated with HPV vaccination among young adults in the United States. Prev Med. (2020) 134:106023. 10.1016/j.ypmed.2020.106023 32061685 PMC7195993

[37] Yoon SJ Park B Kwon J Lim CS Shin YC Jung W. Development of nomograms for predicting prognosis of pancreatic cancer after pancreatectomy: a multicenter study. Biomedicines. (2022) 10:1341. 10.3390/biomedicines10061341 35740364 PMC9220008

[38] Zhang LL Xu F Song D Huang MY Huang YS Deng QL Li YY. Development of a nomogram model for treatment of nonmetastatic nasopharyngeal carcinoma. JAMA Netw Open. (2020) 3:e2029882. 10.1001/jamanetworkopen.2020.29882 33306119 PMC7733160

[39] Fusar-Poli P Hijazi Z Stahl D Steyerberg EW. The science of prognosis in psychiatry: a review. JAMA Psychiatry. (2018) 75:1289–97. 10.1001/jamapsychiatry.2018.2530 30347013

[40] Meehan AJ Lewis SJ Fazel S Fusar-Poli P Steyerberg EW Stahl D. Clinical prediction models in psychiatry: a systematic review of two decades of progress and challenges. Mol Psychiatry. (2022) 27:2700–8. 10.1038/s41380-022-01528-4 35365801 PMC9156409

[41] Fusar-Poli P Stringer D. M S Durieux A Rutigliano G Bonoldi I De Micheli A Stahl D. Clinical-learning vs. machine-learning for transdiagnostic prediction of psychosis onset in individuals at-risk. Transl Psychiatry. (2019) 9:259. 10.1038/s41398-019-0600-9 31624229 PMC6797779

