# Supplementary material for: Factors affecting HPV infection in U.S. and Beijing females: A modeling study
Source: Front Public Health. 2022 Dec 14;10:1052210. doi: 10.3389/fpubh.2022.1052210 (PMC9794849; doi:10.3389/fpubh.2022.1052210)

*Supplementary Materials*

Supplementary Table S1: [Contents of the questionnaire and HPV genotyping.](#TableS1)

Supplementary Table S4: [R packages used in current study.](#TableS4)

Supplementary Table S5: [Characteristics of NHANES cohort.](#TableS5)

Supplementary Table S6: [Characteristics of Beijing female cohort.](#TableS6)

Supplementary Figure S1: [ROC, calibration, and DCA curves of prediction models developed on Modelbest and multivariate logistic regression in NHANES training and test-evaluation sets.](#FigureS1)

Supplementary Figure S2: [ROC, calibration, and DCA curves of prediction models developed on Modelbest and multivariate logistic regression in NHANES test and evaluation sets.](#FigureS2)

Supplementary Figure S3: [ROC, calibration, and DCA curves of prediction models developed on Modelbest and multivariate logistic regression in Beijing female training and test-evaluation sets.](#FigureS3)

Supplementary Figure S4: [ROC, calibration, and DCA curves of prediction models developed on Modelbest and multivariate logistic regression in Beijing female test and evaluation sets.](#FigureS4)

**Supplementary Table S1**. Contents of the questionnaire and HPV genotyping.

| Variables in NHANES females | Variable definition | Variables in Beijing females | Variable definition |
| --- | --- | --- | --- |
| ◆HPV genotype (LBDRPCR) including 37 HPV types: 6, 11, 16, 18, 26, 31, 33, 35, 39, 40, 42, 45, 51, 52, 53, 54, 55, 56, 58, 59, 61, 62, 64, 66, 67, 68, 69, 70, 71, 72, 73, 81, 82, 83, 84, 89, and IS39. | Negative; Positive | ◆HPV genotype including 15 HPV types: 6, 11, 16, 18, 31, 33, 35, 39, 45, 51, 52, 56, 58, 59, 68. | Negative; Positive |
| ◆Age (RIDAGEYR) | 20‒29y; 30‒39y; 40‒49y; 50‒59y | ◆Age | 20‒29y; 30‒39y; 40‒49y; 50‒59y; ≥ 60y |
| ◆Age at first sex (SXD031) | < 18y; ≥ 18y | ◆Age at first sex | < 20y; 20‒29y; 30‒39y |
| ◆Number of male sex partners (SXQ101, SXD101) | 1; ≥ 2 | ◆Number of male sex partners | 1; ≥ 2 |
| ◆Number of previous pregnancies (RHQ160) | ≤ 1; 2; ≥ 3 | ◆Number of previous pregnancies | ≤ 1; 2; ≥ 3 |
| ◆Number of vaginal deliveries (RHQ166) | < 2; ≥ 2 | ◆Number of deliveries | ≤ 1; ≥ 2 |
| ◆Menstruation status (RHQ031) | Regular; Irregular | ◆Menstruation status | Regular; Irregular |
| ◆Times had sex without condom per year (SXQ251) | Never; Always/some times | ◆Main contraceptive measures | Condom; Others |
| ◆Marital status (DMDMARTL) | Married/cohabiting; Living alone (includes never married, divorced, separated and widowed) | ◆Marital status | Married/cohabiting; Living alone (includes never married, divorced, separated and widowed) |
| ◆Education levels (DMDEDUC2) | Less than 9th grade; 9-11th grade (includes 12th grade with no diploma); High school Grad/GED or equivalent; Some college or AA degree; College graduate or above | ◆Education levels | Bachelor or above; Below bachelor |
| ◆Smoking status (SMQ040, SMQ020) | Non-current smoker (including never smoker who denied smoking at least 100 cigarettes and former smoker who is not an active smoker but smoked at least 100 cigarettes in life); Current smoker | ◆Smoking status | Non-current smoker; Current smoker |
| ◆History of HPV infection (SXQ753) | No; Yes | ◆History of HPV infection | No; Yes |
| ◆History of chlamydia (SXQ272) | No; Yes | ◆History of cervical leisions | No; Yes |
| ◆HPV vaccination status (IMQ040, IMQ060) | No; Yes | ◆HPV vaccination status | No; Yes |
| ◆History of trouble sleeping (SLQ050) | No; Yes | ◆Knowledge about HPV infection route | No; Yes |
| ◆Drinking status (ALQ130) | Light drinking (i.e., ≤ 1 drinks per day); Heavy/Binge drinking (i.e., ≥ 2 drinks per day) | ◆Knowledge about HPV prevention | No; Yes |
| ◆Race (RIDRETH1) | Non-Hispanic White; Mexican American; Non-Hispanic Black; Other Hispanic; Other race | ◆Hygiene practices during sex | No; Yes |
| ◆Annual family income (INDFMIN2) | < $20,000; ≥ $20,000 | ◆Regular cervical cancer screening | No; Yes |
| ◆Insurance (HIQ011) | No; Yes |  |  |
| ◆Sedentary activity per day (PAD680) | < 10h; ≥ 10h |  |  |

The codes of the variables in NHANES database are in parentheses of the first column. HPV, Human Papillomaviruses; NHANES, National Health and Nutrition Examination Survey.

**Supplementary Table S4.** R packages used in current study.

| R Package name | Application |
| --- | --- |
| Modelbest (v. 0.1.0) | Choose the optimal prediction model. |
| glmnet (v.4.1-3) | LASSO regression analysis |
| rms (v.6.2-0) | Multivariable logistic regression analysis |
| forestplot (v.2.0.1) | Visualization of forest plots |
| rms (v.6.2-0) and regplot (v. 1.1) | Obtain nomogram |
| DynNom (v. 5.0.1), shiny (v. 1.7.1) and rsconnect (v. 0.8.25) | Obtain online calculator |
| rms (v.6.2-0) and ROCR (v. 1.0-11) | Obtain receiver operating characteristic curve (ROC) with area under the curve (AUC) |
| rms (v.6.2-0) and Hmisc (v. 4.6-0) | Obtain Harrells concordance index (C-index) |
| rms (v.6.2-0) and PredictABEL (v. 1.2-4) | Obtain net reclassification index (NRI) and integrated discrimination index (IDI) |
| rms (v.6.2-0) | Obtain calibration curve |
| rms (v.6.2-0) and rmda (v. 1.6) | Decision curve analysis (DCA) |

**Supplementary Table S5.** General characteristics of females from NHANES.

| Variables | Total  (n = 2259) | Training set  (n = 1581) | Test-Evaluation set  (n = 678) | *P* value |
| --- | --- | --- | --- | --- |
| HPV phenotyping results, n (%) | | | | |
| Negative | 1226 (54.3) | 842 (53.3) | 384 (56.6) | 0.152 |
| Positive | 1033 (45.7) | 739 (46.7) | 294 (43.4) |  |
| HPV vaccination status, n (%) | | | | |
| No | 2075 (91.9) | 1451 (91.8) | 624 (92) | 0.903 |
| Yes | 184 (8.1) | 130 (8.2) | 54 (8) |  |
| Demographic characteristics | | | | |
| Age, n (%) |  |  |  |  |
| 20-29 y | 488 (21.6) | 339 (21.4) | 149 (22) | 0.823 |
| 30-39 y | 718 (31.8) | 503 (31.8) | 215 (31.7) |  |
| 40-49 y | 686 (30.4) | 475 (30) | 211 (31.1) |  |
| 50-59 y | 367 (16.2) | 264 (16.7) | 103 (15.2) |  |
| Race, n (%) |  |  |  |  |
| Non-Hispanic White | 928 (41.1) | 651 (41.2) | 277 (40.9) | 0.528 |
| Mexican American | 349 (15.4) | 239 (15.1) | 110 (16.2) |  |
| Non-Hispanic Black | 525 (23.2) | 368 (23.3) | 157 (23.2) |  |
| Other Hispanic | 232 (10.3) | 172 (10.9) | 60 (8.8) |  |
| Other Race | 225 (10) | 151 (9.6) | 74 (10.9) |  |
| Annual family income, n (%) |  |  |  |  |
| < $20000 | 464 (20.5) | 323 (20.4) | 141 (20.8) | 0.888 |
| ≥ $20000 | 1795 (79.5) | 1258 (79.6) | 537 (79.2) |  |
| Insurance, n (%) |  |  |  |  |
| No | 539 (23.9) | 394 (24.9) | 145 (21.4) | 0.080 |
| Yes | 1720 (76.1) | 1187 (75.1) | 533 (78.6) |  |
| Marital status, n (%) |  |  |  |  |
| Married/cohabiting | 1492 (66) | 1033 (65.3) | 459 (67.7) | 0.300 |
| Living alone | 767 (34) | 548 (34.7) | 219 (32.3) |  |
| Education levels, n (%) |  |  |  |  |
| Less than 9th grade | 94 (4.2) | 62 (3.9) | 32 (4.7) | 0.716 |
| 9-11th grade (includes 12th grade with no diploma) | 269 (11.9) | 195 (12.3) | 74 (10.9) |  |
| High school Grad/GED or equivalent | 459 (20.3) | 326 (20.6) | 133 (19.6) |  |
| Some college or AA degree | 816 (36.1) | 570 (36.1) | 246 (36.3) |  |
| College graduate or above | 621 (27.5) | 428 (27.1) | 193 (28.5) |  |
| Menstrual and reproductive history | | | | |
| Number of previous pregnancies, n (%) |  |  |  |  |
| ≤ 1 | 405 (17.9) | 272 (17.2) | 133 (19.6) | 0.323 |
| 2 | 603 (26.7) | 420 (26.6) | 183 (27) |  |
| ≥ 3 | 1251 (55.4) | 889 (56.2) | 362 (53.4) |  |
| Number of vaginal deliveries, n (%) |  |  |  |  |
| < 2 | 1094 (48.4) | 761 (48.1) | 333 (49.1) | 0.703 |
| ≥ 2 | 1165 (51.6) | 820 (51.9) | 345 (50.9) |  |
| Menstruation status, n (%) |  |  |  |  |
| Regular | 1863 (82.5) | 1294 (81.8) | 569 (83.9) | 0.259 |
| Irregular | 396 (17.5) | 287 (18.2) | 109 (16.1) |  |
| History of infections | | | | |
| History of HPV infection, n (%) |  |  |  |  |
| No | 2023 (89.6) | 1415 (89.5) | 608 (89.7) | 0.960 |
| Yes | 236 (10.4) | 166 (10.5) | 70 (10.3) |  |
| History of chlamydia, n (%) |  |  |  |  |
| No | 2218 (98.2) | 1552 (98.2) | 666 (98.2) | 1.000 |
| Yes | 41 (1.8) | 29 (1.8) | 12 (1.8) |  |
| Sexual lifestyles | | | | |
| Age at first sex, n (%) |  |  |  |  |
| < 18 y | 1473 (65.2) | 1049 (66.4) | 424 (62.5) | 0.090 |
| ≥ 18 y | 786 (34.8) | 532 (33.6) | 254 (37.5) |  |
| Number of male sex partners, n (%) |  |  |  |  |
| 1 | 266 (11.8) | 185 (11.7) | 81 (11.9) | 0.925 |
| ≥ 2 | 1993 (88.2) | 1396 (88.3) | 597 (88.1) |  |
| Times had sex without condom per year, n (%) |  |  |  |  |
| Never | 519 (23) | 370 (23.4) | 149 (22) | 0.494 |
| Always/some times | 1740 (77) | 1211 (76.6) | 529 (78) |  |
| Other lifestyles | | | | |
| Smoking status, n (%) |  |  |  |  |
| Non-current smoker | 1710 (75.7) | 1200 (75.9) | 510 (75.2) | 0.770 |
| Current smoker | 549 (24.3) | 381 (24.1) | 168 (24.8) |  |
| Drinking status, n (%) |  |  |  |  |
| Light drinking | 825 (36.5) | 582 (36.8) | 243 (35.8) | 0.695 |
| Heavy/binge drinking | 1434 (63.5) | 999 (63.2) | 435 (64.2) |  |
| Sedentary activity per day, n (%) |  |  |  |  |
| < 10 h | 1823 (80.7) | 1284 (81.2) | 539 (79.5) | 0.374 |
| ≥ 10 h | 436 (19.3) | 297 (18.8) | 139 (20.5) |  |
| History of trouble sleeping, n (%) |  |  |  |  |
| No | 1648 (73) | 1144 (72.4) | 504 (74.3) | 0.359 |
| Yes | 611 (27) | 437 (27.6) | 174 (25.7) |  |

Q1, first quartile; Q3, third quartile.

**Supplementary Table S6.** General characteristics of females from Beijing.

| Variables | Total  (n = 1593) | Training set  (n = 1115) | Test-Evaluation set  (n = 478) | *P* value |
| --- | --- | --- | --- | --- |
| HPV phenotyping results, n (%) | | | | |
| Negative | 1462 (91.8) | 1030 (92.4) | 432 (90.4) | 0.218 |
| Positive | 131 (8.2) | 85 (7.6) | 46 (9.6) |  |
| HPV vaccination status, n (%) | | | | |
| No | 1511 (94.9) | 1062 (95.2) | 449 (93.9) | 0.335 |
| Yes | 82 (5.1) | 53 (4.8) | 29 (6.1) |  |
| Demographic characteristics | | | | |
| Age, n (%) |  |  |  |  |
| 20‒29 y | 106 (6.7) | 78 (7) | 28 (5.9) | 0.728 |
| 30‒39 y | 540 (33.9) | 384 (34.4) | 156 (32.6) |  |
| 40‒49 y | 574 (36) | 401 (36) | 173 (36.2) |  |
| 50‒59 y | 242 (15.2) | 164 (14.7) | 78 (16.3) |  |
| ≥ 60 y | 131 (8.2) | 88 (7.9) | 43 (9) |  |
| Marital status, n (%) |  |  |  |  |
| Married/cohabiting | 1555 (97.6) | 1095 (98.2) | 460 (96.2) | 0.029 |
| Living alone | 38 (2.4) | 20 (1.8) | 18 (3.8) |  |
| Education levels, n (%) |  |  |  |  |
| Bachelor or above | 1044 (65.5) | 742 (66.5) | 302 (63.2) | 0.216 |
| Below bachelor | 549 (34.5) | 373 (33.5) | 176 (36.8) |  |
| Menstrual and reproductive history | | | | |
| Number of previous pregnancies, n (%) |  |  |  |  |
| ≤ 1 | 660 (41.4) | 471 (42.2) | 189 (39.5) | 0.555 |
| 2 | 504 (31.6) | 345 (30.9) | 159 (33.3) |  |
| ≥ 3 | 429 (26.9) | 299 (26.8) | 130 (27.2) |  |
| Number of deliveries, n (%) |  |  |  |  |
| ≤ 1 | 1382 (86.8) | 958 (85.9) | 424 (88.7) | 0.155 |
| ≥ 2 | 211 (13.2) | 157 (14.1) | 54 (11.3) |  |
| Menstruation status, n (%) |  |  |  |  |
| Regular | 961 (60.3) | 673 (60.4) | 288 (60.3) | 1.000 |
| Irregular | 632 (39.7) | 442 (39.6) | 190 (39.7) |  |
| History of cervical leisions and infections | | | | |
| History of cervical leisions, n (%) |  |  |  |  |
| No | 1298 (81.5) | 907 (81.3) | 391 (81.8) | 0.886 |
| Yes | 295 (18.5) | 208 (18.7) | 87 (18.2) |  |
| History of HPV infection, n (%) |  |  |  |  |
| No | 1466 (92) | 1019 (91.4) | 447 (93.5) | 0.182 |
| Yes | 127 (8) | 96 (8.6) | 31 (6.5) |  |
| Sexual lifestyles | | | | |
| Age at first sex, n (%) |  |  |  |  |
| < 20 y | 88 (5.5) | 69 (6.2) | 19 (4) | 0.106 |
| 20‒29 y | 1446 (90.8) | 1009 (90.5) | 437 (91.4) |  |
| 30‒39 y | 59 (3.7) | 37 (3.3) | 22 (4.6) |  |
| Number of male sex partners, n (%) |  |  |  |  |
| 1 | 1496 (93.9) | 1049 (94.1) | 447 (93.5) | 0.750 |
| ≥ 2 | 97 (6.1) | 66 (5.9) | 31 (6.5) |  |
| Main contraceptive measures, n (%) |  |  |  |  |
| Condom | 937 (58.8) | 658 (59) | 279 (58.4) | 0.854 |
| Others | 656 (41.2) | 457 (41) | 199 (41.6) |  |
| Hygiene practices during sex, n (%) |  |  |  |  |
| No | 59 (3.7) | 39 (3.5) | 20 (4.2) | 0.603 |
| Yes | 1534 (96.3) | 1076 (96.5) | 458 (95.8) |  |
| Other lifestyles | | | | |
| Smoking status, n (%) |  |  |  |  |
| Non-current smoker | 1496 (93.9) | 1047 (93.9) | 449 (93.9) | 1.000 |
| Current smoker | 97 (6.1) | 68 (6.1) | 29 (6.1) |  |
| Regular cervical cancer screening, n (%) |  |  |  |  |
| No | 444 (27.9) | 326 (29.2) | 118 (24.7) | 0.073 |
| Yes | 1149 (72.1) | 789 (70.8) | 360 (75.3) |  |
| Knowledge about HPV | | | | |
| Knowledge about HPV infection route, n (%) |  |  |  |  |
| No | 951 (59.7) | 666 (59.7) | 285 (59.6) | 1.000 |
| Yes | 642 (40.3) | 449 (40.3) | 193 (40.4) |  |
| Knowledge about HPV prevention, n (%) |  |  |  |  |
| No | 964 (60.5) | 680 (61) | 284 (59.4) | 0.594 |
| Yes | 629 (39.5) | 435 (39) | 194 (40.6) |  |

**Supplementary Figure S1.** ROC, calibration, and DCA curves of prediction models developed on Modelbest and multivariate logistic regression in NHANES training and test-evaluation sets.


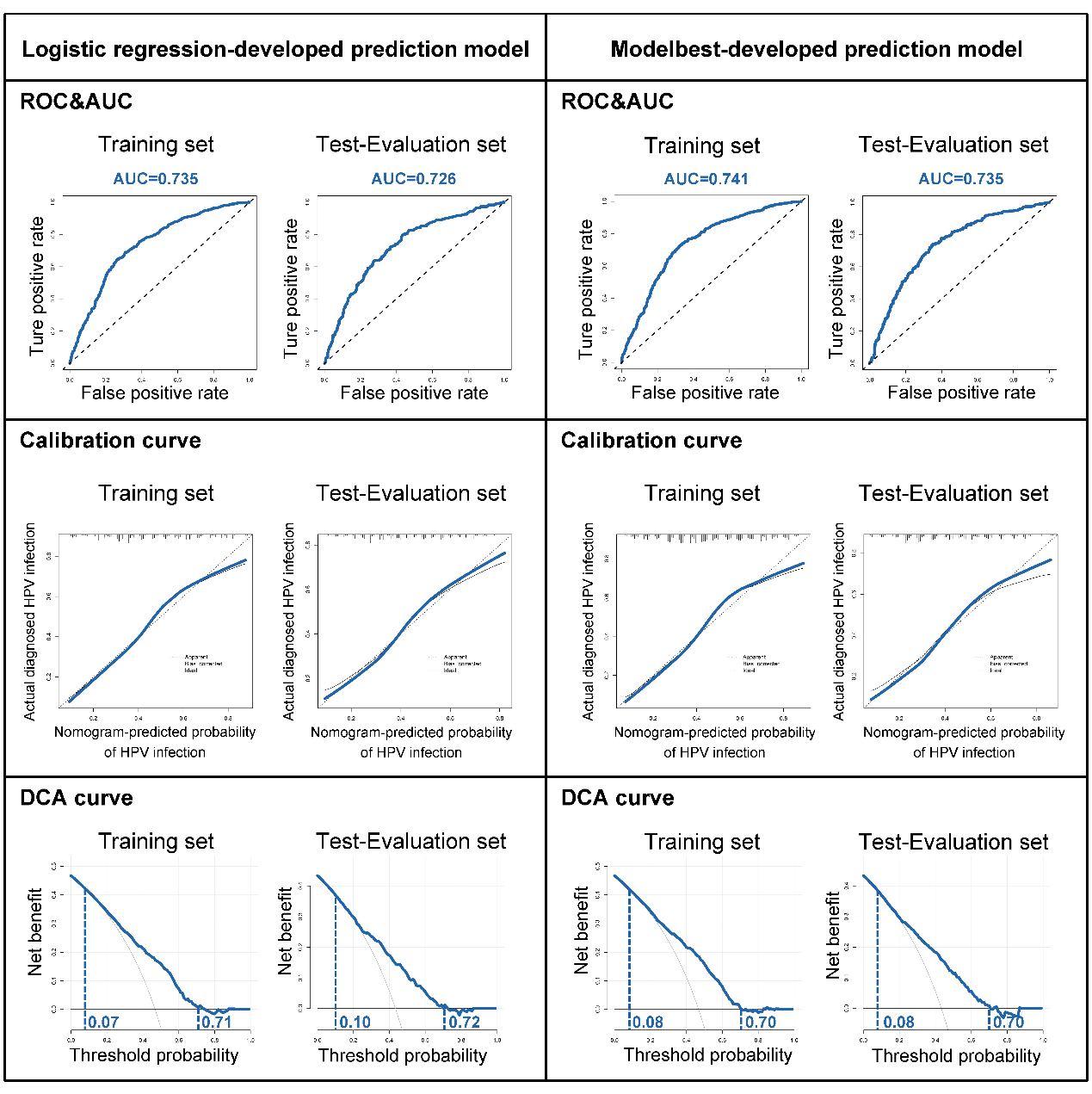


ROC, receiver operating characteristics; DCA, decision curve analysis.

**Supplementary Figure S2.** ROC, calibration, and DCA curves of prediction models developed on Modelbest and multivariate logistic regression in NHANES test and evaluation sets.


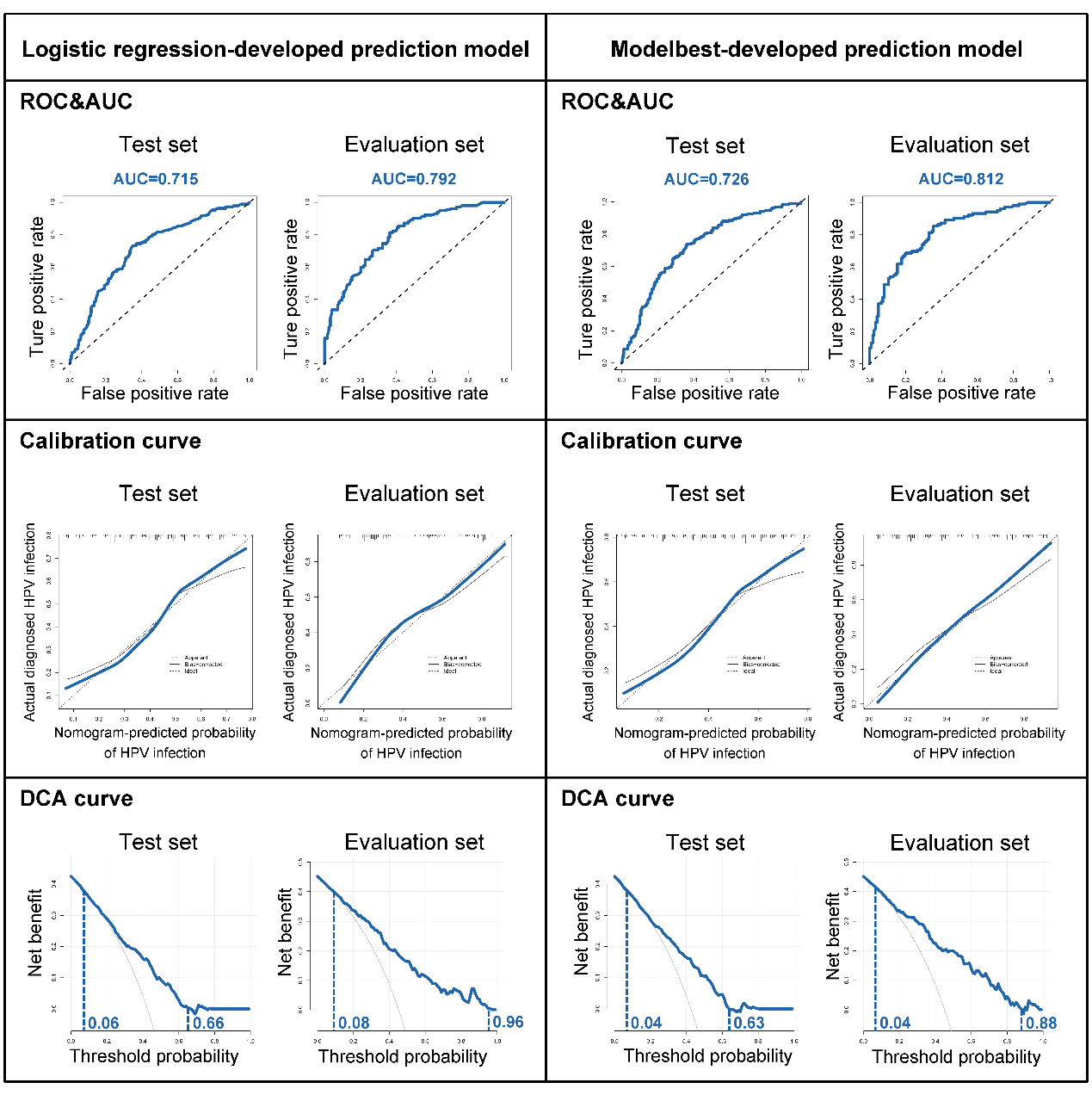


**Supplementary Figure S3.** ROC, calibration, and DCA curves of prediction models developed on Modelbest and multivariate logistic regression in Beijing female training and test-evaluation sets.


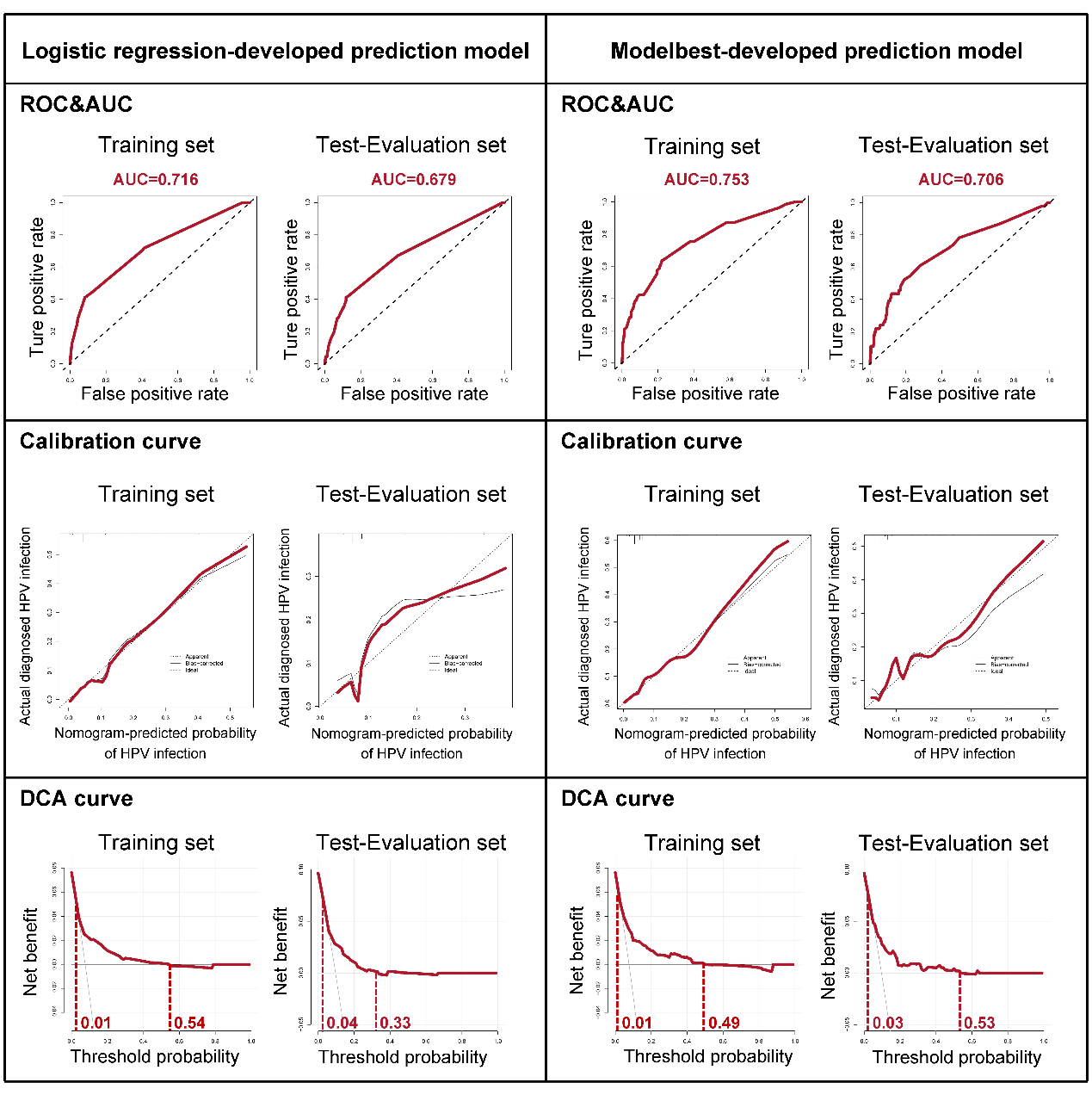


**Supplementary Figure S4.** ROC, calibration, and DCA curves of prediction models developed on Modelbest and multivariate logistic regression in Beijing female test and evaluation sets.


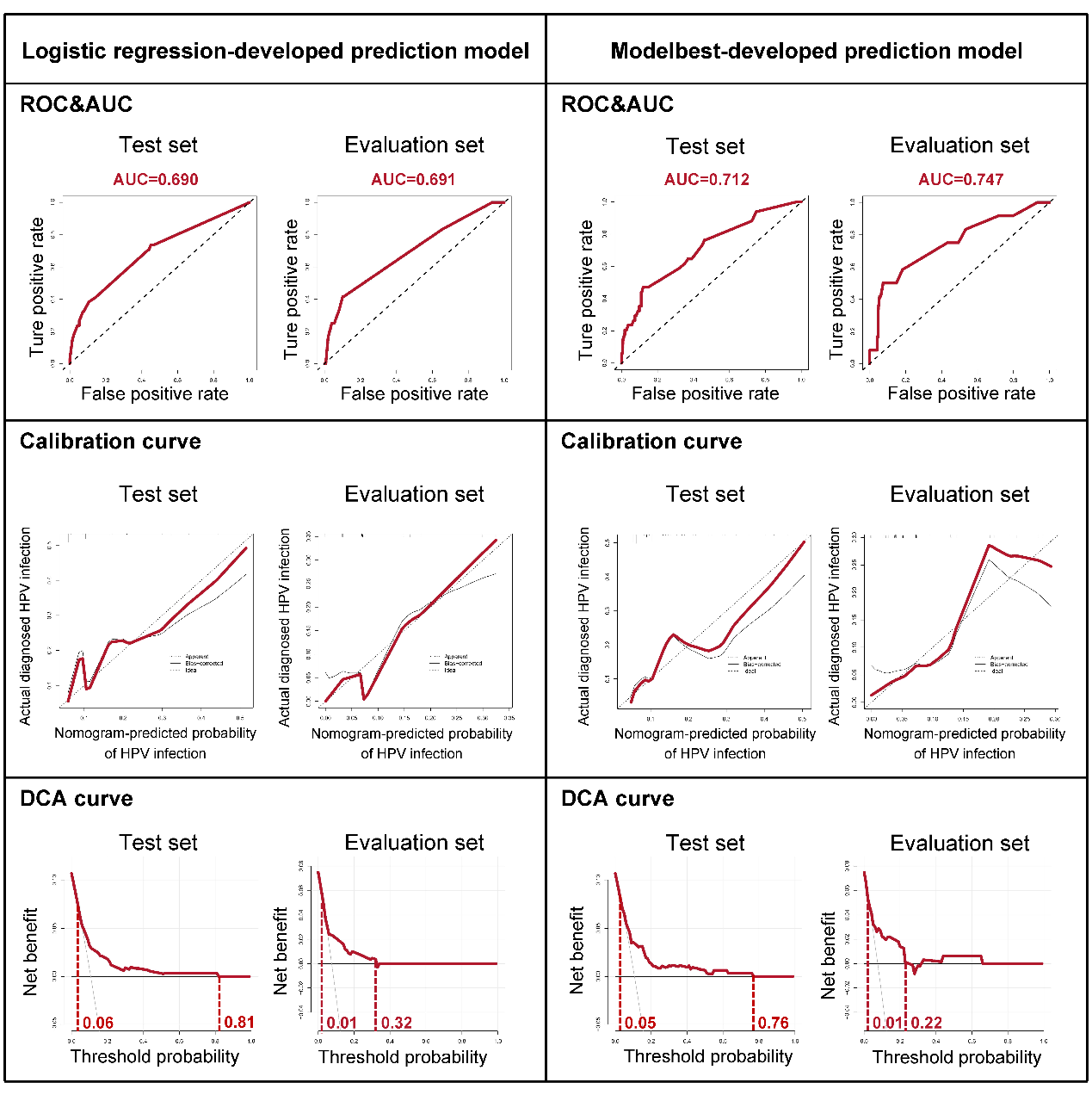

Supplement: Supplementary file 1 [file Table_1.DOCX]
